# Oncological Impact of M-Tor Inhibitor Immunosuppressive Therapy after Liver Transplantation for Hepatocellular Carcinoma: Review of the Literature

**DOI:** 10.3389/fphar.2016.00387

**Published:** 2016-10-21

**Authors:** Giuseppe Tarantino, Paolo Magistri, Roberto Ballarin, Raffaele Di Francia, Massimiliano Berretta, Fabrizio Di Benedetto

**Affiliations:** ^1^Hepato-Pancreato-Biliary Surgery and Liver Transplantation Unit, University of Modena and Reggio EmiliaModena, Italy; ^2^Department of Medical and Surgical Sciences and Translational Medicine, Sapienza – University of RomeRome, Italy; ^3^Hematology, National Cancer Institute, Fondazione “G. Pascale”Napoli, Italy; ^4^Department of Medical Oncology A, National Cancer Institute of AvianoAviano, Italy

**Keywords:** hepatocellular carcinoma, HCC, liver transplantation, mTOR, mTORi, Everolimus, temsirolimus

## Abstract

**Background:** Hepatocellular Carcinoma (HCC) represents the fifth most common malignancy and the third cancer-related cause of death worldwide. Hepatitis B (HBV) and C (HCV) viral infections and alcohol abuse are the principal etiological factors for HCC. Liver transplantation (LT) is oncologically the preferable approach to HCC, as it can remove all the intrahepatic tumor foci, and also the oncogenic cirrhotic liver. The use of mTOR inhibitors (mTORi) for immunosuppression after LT for HCC has been proposed due to rapamycin antitumor activity. We decided to review the literature to clarify the oncological role of mTORi after liver transplantation for HCC, analyzing both present condition and future perspectives.

**Material and Methods:** A systematic literature search was performed using PubMed, EMBASE, Scopus, and the Cochrane Library Central. The search was limited to studies in humans and to those reported in the English language in the period of time between January 2005 and December 2015.

**Results:** The literature search yielded 93 articles; after duplicates were removed, 77 titles and abstracts were reviewed. Most relevant data and papers are herein reported and discussed.

**Conclusions:** So far, the use of mTORi is encouraging in terms of oncological outcomes for patients underwent LT for HCC, both for prevention and treatment of HCC recurrence although definitive data are still awaited.

## Introduction

Hepatocellular Carcinoma (HCC) is the third cancer-related cause of death worldwide and the fifth most common malignancy (Tejeda-Maldonado et al., 2015). During the last 30 years the incidence has almost tripled in the US, and HCC showed to be the fastest rising cause of cancer-related deaths (El-Serag and Kanwal, 2014). HCC is a global burden and prevalence varies worldwide: the incidence is reported to be higher in Asia (accounting for more than 20 cases/100,000) than in North America and Europe (with < 5 cases/100,000) (Tejeda-Maldonado et al., 2015). The principal etiological factors for HCC are hepatitis B (HBV) and C (HCV) viral infections and alcohol abuse, with different age peaks of exposure and various oncological mechanisms requiring different times for hepatocarcinogenesis (Di Benedetto et al., 2013). Metabolic syndrome, obesity, diabetes, aflatoxin B1, tobacco use, coffee consumption, oral contraceptives, and betel quid are other proven risk factors of HCC (Blonski et al., 2010; El-Serag and Kanwal, 2014). Strict surveillance of the “at-risk” population is the only way to achieve an early detection and diagnose HCC when curative treatments are still feasible (de Lope et al., 2012). Ultrasonography (US), is the recommended test for surveillance, with a sensitivity of 65–80% and a specificity >90% (de Lope et al., 2012). The most used staging system for the stratification of HCC severity and consequent treatments is the Barcelona Clinic Liver Cancer staging system (BCLC) (Llovet et al., 1999). BCLC is a multidimensional platform based on (1) patient's performance status, (2) liver function calculated using the Child-Turcotte-Pugh (CTP) score, and (3) tumor's dimension. According to that system HCCs are allocated in five categories. Very early (0) and Early stages (BCLC A) are amenable of curative treatments, such as tumor resection, liver transplantation (LT) and tumor ablation. On the other hand, Intermediate stage (BCLC B) can benefit from Trans-arterial chemoembolization (TACE), while Advanced stage (BCLC C) can be treated with Sorafenib (Llovet et al., 2008). Lastly, Terminal stage (BCLC D) requires only supportive care (Tejeda-Maldonado et al., 2015). Interestingly, BCLC is not a rigid platform: it has to be used considering that a patient being evaluated for therapy may move from an early stage to an intermediate or advanced, because of specific patient profile that may contraindicate the initially proposed treatment, reflecting the “treatment stage migration” concept (de Lope et al., 2012). Moreover, therapeutic approach should always be multidisciplinary and patient-tailored to offer the best treatment possible, due to the multiform essence and the intricate background of HCC development (Berretta et al., 2011; Di Benedetto et al., 2011a). Although liver resection is always the first therapeutic approach that should be considered, LT is oncologically preferable, since it can remove all the intrahepatic localizations of the tumor and the oncogenic cirrhotic liver (de Lope et al., 2012). The application of the Milan criteria (presence of one HCC nodule smaller than 5 cm or a maximum of 3 nodules smaller than 3 cm, without vascular invasion or extra hepatic spread; Mazzaferro et al., 1996) in the selection of patients amenable to receive LT, is related to the best outcomes, with the 5-year survival rate higher than 70% and similar to non-HCC recipients, while recurrence rate ranges from 5 to 15% (de Lope et al., 2012; Mancuso and Perricone, 2014). An adequate immunosuppressive regimen is needed after LT, and chronic kidney disease (CKD), recurrence of HCV and recurrence of HCC are major issues in post-operative management (Kawahara et al., 2011). The first immunosuppressive compounds that provided marked improvements in patient and graft survival after LT were the calcineurin inhibitors (CNIs). Unfortunately, those compounds can frequently cause neurotoxicity, adverse impacts on cardiac risk profile, and more frequently nephrotoxicity, together with risks for malignancy (Kawahara et al., 2011). The research for immunosuppressive agents that minimize the risk of HCC recurrence led to the application of the inhibitors of the mammalian target of rapamycin (mTORi). mTOR is a serine/threonine protein kinase downstream of the phophoinositide-3-kinase (PI3K)-related kinase family that regulates several oncogenic processes, like cellular growth and proliferation, and angiogenesis. Moreover, recent data suggest that mTOR deregulation modulates hepatocellular carcinogenesis (Di Benedetto et al., 2011b; Ashworth and Wu, 2014). In particular, an altered expression of the mTOR pathway have been reported in 40–50% of HCCs, while the activation of the mTOR pathway is related to the presence of less differentiated tumors, earlier recurrence, and worse survival outcomes (Ashworth and Wu, 2014). The anticancer activity of mTORi has also been demonstrated in breast cancer, urinary bladder cancer, and kidney cancer (Lee et al., 2015; Kajiwara and Masuda, 2016; Pinto-Leite et al., 2016). In addition, incidence and speed of HCC recurrence in multivariate analysis are related to mTOR signaling activation in a statistically significant fashion (Duvoux and Toso, 2015). According to recent data, HCC recurrence rate among patients transplanted for HCC within Milan criteria is lower in patients treated with mTORi, compared with those that received CNIs (*p* = 0.03). Moreover, patients transplanted for HCC within Milan criteria show lower HCC recurrence rate when compared with those transplanted for HCC outside Milan criteria, in a statistically significant fashion (Cholongitas et al., 2014b). However, those data reported by Chologitas and colleagues in their review are biased by the differences in risk factors for HCC recurrence: in particular, the percentage of patients treated with mTORi that had been transplanted for HCC within Milan criteria (69%) was significantly lower than the one observed in patients treated with CNIs (74%) (*p* = 0.04) (Cholongitas et al., 2014b). Given Everolimus antitumor activity, it was tested in patients who do not respond to Sorafenib. Unfortunately, the results from a phase III trial comparing Everolimus 7.5 mg daily with placebo (EVOLVE-1 study) declared the failure of Everolimus with non-improvement of overall survival (OS) in advanced HCC patients failed with or intolerant to Sorafenib (Zhu et al., 2014). In detail, this study showed that the median OS in the Everolimus arm was 7.56 months vs. 7.33 months in the placebo arm (*p* = 0.675). The median time to progression (TTP) was 2.96 months vs. 2.6 months (Everolimus vs. placebo). Therefore, no benefit in the median TTP, in the overall population or in any of the pre-stratified subgroups was demonstrated (Zhu et al., 2014; Chuma et al., 2015; Deng et al., 2015; Palmer and Johnson, 2015).

Given the recent results, we decided to review the literature to clarify the oncological role of mTORi after liver transplantation for HCC, analyzing both present condition and future perspectives.

## Materials and methods

### Literature search

PRISMA statement guidelines for conducting and reporting systematic reviews were followed as previously reported.

Two of the manuscript's authors (PM and GT) performed a systematic literature search in the following databases: PubMed, EMBASE, Scopus, and the Cochrane Library Central. The search was limited to studies in humans and to those reported in the English language in the period of time between January 2005 and December 2015. No restrictions were set for the type of publication.

**Figure 1:**
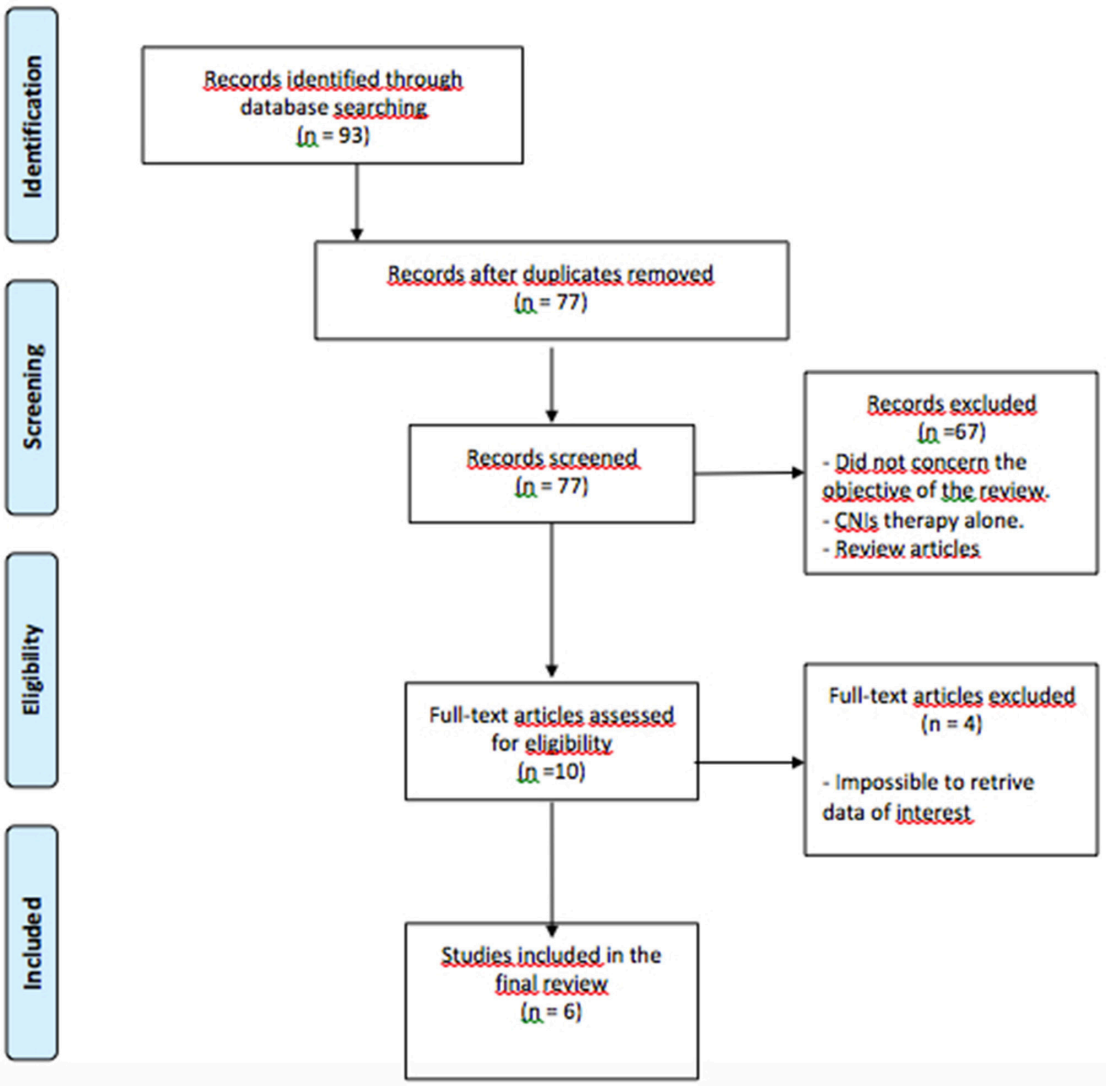
**Flow-chart of the review process**.

The following MESH search headings were used: “hepatocellular carcinoma” OR “hcc” OR “hepatoma” AND “liver transplantation” OR “liver transplant” AND “mTORi” OR “mTOR” OR “mTOR inhibitor” OR “everolimus” OR “temsirolimus.” The reference lists of all retrieved articles fulfilling the inclusion criteria were crosschecked extensively to further enrich the search. We run the last search on December 31th 2015 in all the databases (Figure 1).

### Study selection

Then the same two authors screened the titles and abstracts of the studies that were selected after the first step. Duplicate studies were excluded. The following criteria were set for inclusion in this review: (1) Studies comparing the oncological outcomes of different immunosuppressive regimens; (2) Studies reporting oncologic outcomes after LT for HCC in patients treated with mTORi; and (3) If more than one study was reported by the same institute, only the most recent or the highest quality study was included.

The following exclusion criteria were set: (1) Original studies assessing the outcome of CNIs therapy alone; (2) Review articles, letters, comments and case reports; and (3) Studies in which it was impossible to retrieve or calculate data of interest.

The literature search yielded 93 articles; after duplicates were removed, 77 titles and abstracts were reviewed. Most relevant data and papers are reported in the results section and later discussed.

### Data extraction

The same two authors extracted the main data as follows: (1) First author, year of publication and study type; (2) Number and characteristics of patients; and (3) Treatment outcomes.

Any discrepancy between the two reviewers was resolved by consensus discussion or with the opinion of the Senior Author (FDB). Results are summarized in Table 1.

**Table 1:** **Outcomes of most relevant papers in literature (R, retrospective; OS, Overall Survival; TDP, time to disease progression; NR, not reported)**.

**Author**	**Year**	**No. of patients**	**Type of study**	**Study interval**	**Study design**	**Major outcomes**
Gomez-Martin	2012	31	R	2008–2010	All patients with a history of recurrent HCC after LT who were being treated with mTOR inhibitors and Sorafenib for a tumor relapse that was not susceptible to locoregional therapy were included	Median OS since the start of the treatment with Sorafenib, median TDP after the initiation of the Sorafenib and median OS after relapse were respectively 19.3, 6.77, and 40.1 m
Cholongitas	2014	183	R	2006–2011	All consecutive adult LT recipients with a CNI-based immunosuppression regimen who were converted to Everolimus treatment were included	Recurrence-free survival rate was higher in the Everolimus group of patients compared to those under CNI (*p* = 0.055)
De Simone	2014	7	R	NR	Patients switched to Everolimus + Sorafenib at any time for HCC recurrence after LT were included	At a median follow-up of 6.5 m 71.4% were alive, 57.1% had tumor progression according to the mRECIST criteria and median time to progression was 3.5 m
Ferreiro	2014	21	R	2005–2010	Long-term survival and cumulative recurrence in high-risk patients receiving Everolimus-based immunosuppression after LT for HCC were compared with an historic control group	5-year survival was 60.2% in the Everolimus group and 32.3% in the control group (*p* = 0.05). Treatment with Everolimus was an independent predictor of longer survival (*p* = 0.02).
Bilbao	2015	477	R	1988–2010	The analysis included all maintenance liver transplant patients for whom Everolimus conversion was initiated before December 2010	Survival after conversion to Everolimus at 3 years were 83.0% for renal dysfunction, 71.1% for *de novo* tumors, and 59.5% for HCC

## Results

### The mTOR pathway

In the mTOR pathway there are two important components: mTOR complex 1 (mTORC1) and mTOR complex 2 (mTORC2) (Kawahara et al., 2011; Ashworth and Wu, 2014). m-TORC1 is activated by several signals, like Growth factors (insulin or IGF-1), various cytokines, co-stimulatory signals, Toll-like receptor (TLR) ligands, cellular energy levels, hypoxia, cellular stress, and DNA damage. In particular, the activation of PI3K determines the downstream phosphorylation of serine/threonine protein kinase (PKB/AKT) at protein residue Thr308 by 3-phosphoinositide-dependent protein kinase-1 (PDK1). Downstream signaling through the effector tuberous sclerosis 1-tuberous sclerosis 2 complex (TSC1-TSC2) finally leads to the activation of mTORC1 (Kawahara et al., 2011; Ashworth and Wu, 2014). Then the activated m-TORC1 regulates protein translation, cell proliferation, angiogenesis and autophagy through its two targets, 70S ribosomal protein S6 kinase (S6K1) and the eukaryotic initiation factor 4E binding protein 1 (4E-BP1), which are also responsible of a negative feedback loop (Ashworth and Wu, 2014). Notably, the inhibition of mTORC1 by rapamycin and its analogs disrupts S6K1-mediated feedback inhibition of PI3K signaling, which allows for increased PKB/AKT phosphorylation. It results in an accumulation of phosphorylated AKT which can then activate alternative pathways to inhibit apoptosis and promote cell proliferation (Ashworth and Wu, 2014). Conversely, m-TORC2 is not inhibited directly by rapamycin, and regulates actin cytoskeletal dynamics through the small GTPase RAS homolog (RHO) and protein kinase C (PKC). Moreover, it indirectly promotes mTORC1 activity through activation of PKB/AKT (Kawahara et al., 2011; Ashworth and Wu, 2014). mTORi are capable of effective immunosuppression by blocking cell cycle progression from G1 into S phase in IL-2 stimulated T cell lymphocytes, and decreasing the proliferation of CD4+ T cells. At the same time, they are responsible for the prevention of hepatocellular tumorogenesis through a potent inhibition of angiogenesis (Niemczyk et al., 2009; Kawahara et al., 2011; Ashworth and Wu, 2014). Temsirolimus, Everolimus, Deferolimus are three analogs of rapamycin modified at C43 to increase solubility and bioavailability that differ for the addition of an ester, ether, or phosphonate group respectively (Kawahara et al., 2011).

### mTORi for prevention of HCC recurrence after LT

Cholongitas and colleagues in 2014 reported the results after conversion to Everolimus in a population of 183 patients underwent LT (Cholongitas et al., 2014a). Twenty-one patients were affected by HCC and treated with Everolimus, and no recurrence occurred according to alpha-fetoprotein levels and radiological imaging (CT and/or MRI). Conversely, in the CNI-historical control group HCC recurrence occurred in 18.5% of the 22 patients after a mean of 49 months (range 6–113) of follow-up. Among those, 3 (75%) were outside the Milan criteria. Authors reported that the Everolimus group of patients had a cumulative HCC recurrence-free survival rate higher than the CNI-treatment group, with a marginal statistical significance (log rank *p* = 0.055).

The study by Schwarz et al. published in 2014 reports the outcomes of six patients underwent major emergency or planned abdominal or thoracic surgical procedures without mTORi discontinuation (Schwarz et al., 2014). Five patients were switched to mTORi due to (1) HCC prevention or recurrence (three patients), (2) *de novo* colorectal cancer (one patient), and (3) posterior reversible encephalopathy syndrome related to Tacrolimus (one patient), while one patient received mTORi as primary immunosuppressive therapy. Two HCC recurrences were observed. The postoperative mortality was nil, and no reoperations were needed. Authors reported no evisceration, incisional surgical site infection, nor lymphocele. Later two patients developed incisional hernias at 12 and 18 months respectively.

Bilbao and colleagues in their paper published in 2015 reported a cohort of 477 patients underwent LT in whom therapy conversion to Everolimus was initiated before 2010 (Bilbao et al., 2015). One hundred and forty five patients received LT for HCC, with a post-operative median follow-up time of 4.5 years (interquartile range, 3–5.9). In their series the conversion to Everolimus was indicated for prophylactic reasons in 100 patients (69.0%) and as a result of tumor recurrence in 38 patients (26.2%) (data not available in the other 7 cases). Among the prophylaxis group, half of the cases were converted to Everolimus treatment within 4 months after LT at a median time of 4 months (interquartile range, 2–12). Similarly, 143 patients underwent LT for non-hepatic *de novo* malignancy: the median follow-up was 9.1 years (interquartile range, 6.93–14), with a prevalence of gastrointestinal and skin tumors. In the overall study population, the mean length of survival was 14.4 years (standard error, 0.5) from LT and 4.5 years (standard error, 0.2) from conversion to Everolimus. The survival rates after LT at 5-, 10-, and 15-years were respectively 78.4, 64.2, and 56.8%. After conversion to Everolimus the survival rate at 1-, 3-, and 5-years were respectively 88.2, 72.1, and 60.8%. Survival rate after conversion to Everolimus at 3-years were 71.1% for *de novo* tumors, and 59.5% for HCC patients. The most frequent cause of death (*n* = 136, 28.5%) was tumor progression (*n* = 68, 14.3%), followed by liver failure (*n* = 18, 3.8%) and infection (*n* = 12, 2.5%).

The study reported by Ferreiro and colleagues in 2015 compared two groups of patients receiving LT for end-stage liver disease and HCC who were deemed to have a high risk of post-transplant recurrence (Ferreiro et al., 2014). The inclusion criteria for the high-risk group were (1) if they exceeded the Milan criteria; (2) if they had macrovascular or microvascular invasion; (3) poorly differentiated tumors in the explanted liver. Twenty-one patients, selected from a total of 72 patients receiving LT from February 2005 to December 2010 were included in the Everolimus group. Conversely, 31 patients, selected from a historic control group of 120 patients receiving LT and treated with CNIs from May 1994 to January 2005 were included in the control group. Two years survival was 85.7% in the Everolimus group compared to 64.5% in the control group, while 5-year survival rates were 60.2 and 32.3%, respectively (*p* = 0.05). Any statistically significance was found in the 2- and 5-year cumulative recurrence rates, reported as 23.8 and 41.3% in the Everolimus group, compared to 45.2 and 61.3%, respectively, in the control group (*p* = 0.17). A subgroup of 32 patients diagnosed with well or moderately differentiated HCC was studied (10 in the Everolimus group and 22 in the control group): in this population, 2-year survival rates was 100 and 68.2%, respectively, and 5-year survival rates were 88.9 and 40.9%, respectively (*p* = 0.02), but any significant difference was observed in cumulative recurrence rates between the two groups (*p* = 0.22). In addition, any significant difference in survival or cumulative recurrence rates was found among patients with poorly differentiated HCC. Finally, in the multivariate analysis, Everolimus treatment resulted as an independent predictor of longer survival (HR. 0.34; 95% CI, 0.14–0.83; *p* = 0.02), while poorly differentiated tumors (HR. 2.77; 95% CI, 1.24–6.15; *p* = 0.01) and >3 nodules (HR. 2.37; 95% CI, 1.07–5.26; *p* = 0.03) were predictors of shorter survival.

### mTORi for treatment of HCC recurrence after LT

In 2012 Gomez-Martin and colleagues reported their experience in a descriptive, open, multicenter, retrospective, uncontrolled cohort study designed to assess the combined use of an mTOR inhibitor (Everolimus or Sirolimus) and Sorafenib in the treatment of patients with HCC recurrence after LT (Gomez-Martin et al., 2012). Thirty-one patients were included: in 30 of the 31 patients Everolimus or Sirolimus was introduced to replace CNI immunosuppressants after the diagnosis of post-transplant HCC recurrence. In detail, 22 patients received Everolimus (mean dose 2.23 mg daily), and 8 received Sirolimus (mean dose 2.63 mg daily), while 1 patient was already receiving Everolimus as immunosuppressive treatment. Twenty-six received a combination treatment with Sorafenib (so called “efficacy group”): 10 patients begun at the full dosage (800 mg/day) while the other 16 patients begun at 400 mg/day. Among those latter cases, 4 did not ever reach the full dosage because of side effects. There was 1 partial response (PR), and 13 cases with disease stabilization as the best response. The reported median overall survival since the start of the treatment with Sorafenib, median time to disease progression after the initiation of the Sorafenib and median overall survival after relapse were respectively 19.3 months (95% CI.13.4–25.1 months), 6.77 months (95% CI. 2.3–11.1 months), and 40.1 months (95% CI. 10.1–70.1 months).

De Simone and colleagues published in 2014 a retrospective study based on a prospectively collected database including LT recipients (De Simone et al., 2014). All the adults recipients of a primary or secondary liver graft, transplanted for HCC within and beyond Milan criteria, who received Everolimus plus Sorafenib for HCC recurrence not amenable to surgical resection and/or locoregional treatment were considered for inclusion. Finally, seven patients fulfilled the inclusion criteria. At a median follow-up of 6.5 months, five patients (71.4%) were alive and four (57.1%) had tumor progression according to the mRECIST criteria, and the median time to progression was 3.5 months (highest interquartile range 12 months). Two patients died at a median follow-up of 5 months (tumor progression in 1 patient and sepsis in the other).

## Discussion

The use of Everolimus in the post-operative course after LT should be adjusted according to evidences reported in literature on side effects and morbidity (Dumortier et al., 2016). In particular, the introduction in the therapeutic regimens should be delayed until complete surgical healing if the patient had surgical complications, infections or a general condition that may negatively affect wound healing, due to the adverse effect of Everolimus on wound repair. Moreover, early introduction of Everolimus should be avoided in malnourished patients, and should be interrupted 4–5 days before major elective surgery. The product license recommends that Everolimus should be initiated ~4 weeks after LT at a starting dose of 1.0 mg twice daily, targeting a trough concentration 3–8 ng/mL, in combination with tacrolimus and steroids. CNI dose should be progressively reduced only after Everolimus trough level reaches the range 3–8 ng/mL, with a final tacrolimus target range of 3–5 ng/mL.

In case of recurrent HCC, Everolimus should be introduced at 1 mg twice each day, with target levels of 8–12 ng/ml according to patient tolerability, and discontinuing CNI as early as possible. A combination therapy with Sorafenib may also be considered. Finally, the RESCUE study demonstrated that Everolimus should be introduced as soon as possible in response to renal disease induced by medical therapy, since a significant inverse relationship between baseline renal function and the renal benefit from switch to Everolimus was showed (Dumortier et al., 2016).

The results reported by Zhu and colleagues in their international double blind placebo-controlled phase 3 study, deeply modify the oncological research approach for advanced HCC (Zhu et al., 2014). However, previous organ transplantation requiring immunosuppression, long-term immunosuppressive regimens, and HIV infection, were all considered as exclusion criteria from their work (Zhu et al., 2014). Therefore, the role of mTORi for HCC recurrence after LT is not predictable by those data and further studies are needed. Currently, the results of an open-labeled, randomized, prospective multicenter trial comparing Sirolimus-containing vs. mTOR-inhibitor-free immunosuppression in patients undergoing LT for HCC are awaited (ClinicalTrial.gov identifier: NCT00355862) (Schnitzbauer et al., 2010). The purpose of this study is to determine the safety and efficacy of this therapeutic regimen in patients following LT for HCC, with regard to HCC recurrence-free patient survival (Schnitzbauer et al., 2010). The use of Sorafenib and mTORi and their combination has been proposed for both neoadjuvant and adjuvant approach for HCC due to their synergistic action (Di Benedetto et al., 2011c, 2012; Zheng et al., 2015). We herein reported the study by Gomez-Martin and colleagues that show why the combination of Sorafenib and mTORi is a valid option for post LT HCC recurrence not candidate to curative treatments (Gomez-Martin et al., 2012). This paper, besides the intrinsic limitations due to its design, raises the problem of toxicity, since it led to dose reductions in 2 of the 23 patients treated with Everolimus and in 4 of the 8 patients treated with Sirolimus. This is consistent with the results by De Simone et al. (2014). In fact, their study confirms the validity of Everolimus and Sorafenib combination, but shows a high toxicity rate. Hand-foot syndrome was observed in five patients (71.4%), hypertension in 1 (14.3%), alopecia in 1 (14.3%), hypothyroidism in 1 (14.3%), diarrhea in 2 (28.6%), pruritus in 1 (14.3%), abdominal pain in 1 (14.3%), rash in 1 (14.3%), asthenia in 3 (42.8%), anorexia in 3 (42.8%), and hoarseness in 2 (28.6%). Moreover, adverse events led to temporary Sorafenib discontinuation in two patients (28.6%) and to dose reduction in three (42.8%) (Gomez-Martin et al., 2012; De Simone et al., 2014). The combined administration of Everolimus and Sorafenib was already tested in the setting of Advanced Clear Cells Renal Carcinoma (RCC) (Harzstark et al., 2011; Amato et al., 2012; Hainsworth et al., 2013). Hainsworth and colleagues (Phase I/II trial) reported an overall response rate of 13% (partial in each case), while in terms of tolerability 11% of patients quit the treatment due to toxicity and 1 death occurred due to intracranial hemorrage (Hainsworth et al., 2013). Harzstark et al (Phase I study) showed in their experience that among 20 patients enrolled in 3 cohorts, 6 achieved a partial response. Notably, the administration of Sorafenib 400 mg twice daily plus Everolimus 5 mg daily, was reported to be the maximum tolerated dose for this regimen. Conversely, Amato and colleagues (Phase 1 trial) concluded that a dose of Everolimus of 10 mg each day plus Sorafenib 400 mg twice-a-day is safe and effective for progressive metastatic RCC since no patient in that cohort developed dose-limited toxicity (Amato et al., 2012). Should be noted that patients candidate for liver transplantation are part of a strictly selected population that fulfill international criteria, as above-mentioned. Conversely, patients with advanced HCC intolerant to Sorafenib or with progressive disease, as well as patients with other solid malignancies with a metastatic diffusion, have a completely different disease that justifies the different therapeutic regimen. As a matter of fact, the use of mTORi in liver transplantation has first of all an immunosuppressant intent, and their use in the post-operative course should be adjusted according to evidences reported in literature to prevent side effects and morbidity.

About the oncological outcomes, Cholongitas and colleagues reported no HCC recurrence in a population of 21 patients converted to Everolimus, after a median follow-up of 48 months (range 12–76). Meanwhile, recurrence rate in patients treated with CNI was higher (0 vs. 18.5%, *p* = 0.055) (Cholongitas et al., 2014a). The recent study by Bilbao et al. shows that, as previously reported (Di Benedetto et al., 2007, 2009), the conversion to Everolimus is related to benefits in terms of improvement to eGFR, regardless of the grade of renal dysfunction (Bilbao et al., 2015). Conversely, oncological outcomes are inconclusive and the study is biased by the retrospective design and the heterogeneity of the patients, which were enrolled from 20 centers. Ferreiro and colleagues reported in their work that Everolimus is an independent predictor of prolonged survival in patients at high risk of HCC recurrence after LT (Ferreiro et al., 2014). Interestingly, in their study the beneficial effect of Everolimus was maintained in patients with well or moderately differentiated HCC but was not observed in those with poorly differentiated tumors. Finally, although not of oncological interest, the results by Schwarz and colleagues showed that treatment with mTORi after LT does not preclude the possibility of further surgical procedures (Schwarz et al., 2014).

## Conclusions

Results in literature are contradictory and the populations examined are too heterogeneous to draw definite conclusions. So far, the use of mTORi is encouraging in terms of oncological outcomes for patients underwent LT for HCC, both for prevention and treatment of HCC recurrence. About mTORi and Sorafenib combination, future studies should critically address the issue of toxicity and the real balance between risks and benefits for patients.

## Author contributions

FD, MB: study conception and design. PM, GT, RB: manuscript drafting. FD, MB: manuscript critical revision. PM, GT: literature review. PM, GT: data extraction. RB, PM, GT: data analysis. All the authors approved and are fully conversant with the final version of the manuscript.

### Conflict of interest statement

None of the Authors, nor the Institutions they represent, at any time received payment or services from a third party for any aspect of this work. Authors have no financial relationships with entities that could be perceived to influence, or that give the appearance of potentially influencing this manuscript. No patents and copyrights, whether pending, issued, licensed and/or receiving royalties are relevant to the work.
